# The pathological effects of CCR2+ inflammatory monocytes are amplified by an IFNAR1-triggered chemokine feedback loop in highly pathogenic influenza infection

**DOI:** 10.1186/s12929-014-0099-6

**Published:** 2014-11-18

**Authors:** Sue-Jane Lin, Ming Lo, Rei-Lin Kuo, Shin-Ru Shih, David M Ojcius, Jean Lu, Chien-Kuo Lee, Hui-Chen Chen, Meei Yun Lin, Chuen-Miin Leu, Chia-Ni Lin, Ching-Hwa Tsai

**Affiliations:** Research Center for Emerging Viral Infections, College of Medicine, Chang Gung University, Tao-Yuan, Taiwan; Graduate institute of Medical Biotechnology, College of Medicine, Chang Gung University, Tao-Yuan, Taiwan; Department of Medical Biotechnology and Laboratory Science, College of Medicine, Chang Gung University, Tao-Yuan, Taiwan; Graduate Institute of Microbiology, College of Medicine, National Taiwan University, Taipei, Taiwan; Department of Molecular Cell Biology, Health Sciences Research Institute, University of California, Merced, CA USA; Genomics Research Center, Academia Sinica, Taipei, Taiwan; Graduate Institute of Immunology, College of Medicine, National Taiwan University, Taipei, Taiwan; Graduate Institute of Basic Medical Science, China Medical University, Taichung, Taiwan; Institute of Microbiology and Immunology, National Yang-Ming University, Taipei, Taiwan; Department of Laboratory Medicine, Chang Gung Memorial Hospital, Linkou, Taiwan

**Keywords:** Influenza A virus, CCR2+ inflammatory monocytes, IFNAR1, CCL2, CCL7 and CCL12

## Abstract

**Background:**

Highly pathogenic influenza viruses cause high levels of morbidity, including excessive infiltration of leukocytes into the lungs, high viral loads and a cytokine storm. However, the details of how these pathological features unfold in severe influenza infections remain unclear. Accumulation of Gr1 + CD11b + myeloid cells has been observed in highly pathogenic influenza infections but it is not clear how and why they accumulate in the severely inflamed lung. In this study, we selected this cell population as a target to investigate the extreme inflammatory response during severe influenza infection.

**Results:**

We established H1N1 IAV-infected mouse models using three viruses of varying pathogenicity and noted the accumulation of a defined Gr1 + CD11b + myeloid population correlating with the pathogenicity. Herein, we reported that CCR2+ inflammatory monocytes are the major cell compartments in this population. Of note, impaired clearance of the high pathogenicity virus prolonged IFN expression, leading to CCR2+ inflammatory monocytes amplifying their own recruitment via an interferon-α/β receptor 1 (IFNAR1)-triggered chemokine loop. Blockage of IFNAR1-triggered signaling or inhibition of viral replication by Oseltamivir significantly suppresses the expression of CCR2 ligands and reduced the influx of CCR2+ inflammatory monocytes. Furthermore, trafficking of CCR2+ inflammatory monocytes from the bone marrow to the lung was evidenced by a CCR2-dependent chemotaxis. Importantly, leukocyte infiltration, cytokine storm and expression of iNOS were significantly reduced in *CCR2−/−* mice lacking infiltrating CCR2+ inflammatory monocytes, enhancing the survival of the infected mice.

**Conclusions:**

Our results indicated that uncontrolled viral replication leads to excessive production of inflammatory innate immune responses by accumulating CCR2+ inflammatory monocytes, which contribute to the fatal outcomes of high pathogenicity virus infections.

## Background

Influenza A virus (IAV) is a common human respiratory virus and causes seasonal epidemic and pandemic infections. In the past 100 years, pandemics of influenza have been caused by the IAV strains H1N1 (1918), H2N2 (1957), H3N2 (1968) and H1N1 (2009) [1,2]. These pandemic strains vary in their virulence and pathogenicy. Compared to the 1968 and 2009 pandemics, the 1957 pandemic featured intermediate pathogenicity, while the virus causing the 1918 pandemic was relatively highly pathogenic in the human population [2]. Currently we are threatened by sporadic infections by emerging avian IAVs, including highly pathogenic avian H5N1 and H7N9 viruses [1,3]. Two well known, highly pathogenic IAVs, 1918 H1N1 and avian H5N1 cause high levels of morbidity including excessive infiltration of neutrophils and monocytes into the lungs, high viral loads and hypercytokinemia, with significant increases of IL-1, IL-6, IL-8, TNF, CXCL10 and CCL2 in the patients’ plasma [4-6]. Thus, cytokines and chemokines induced at high levels by IAV infections have become targets for the development of IAV therapy. However, the results of experiments using knock-out mice indicate that none of them alone determines highly pathogenic virus-induced lethality [7,8]. Thus, we used another approach to identify the immune cell types that are recruited during infection and contribute to the excessive inflammatory responses during highly pathogenic virus infection.

H1N1 IAV circulate continuously in the human population, and the three H1N1 strains selected for this study display low, intermediate and high virulence in mice as follows: (1) seasonal H1N1 A/Taiwan/141/02 (141; low virulence); (2) pandemic H1N1 A/Taiwan/126/2009 (swine-origin influenza virus, SOIV; intermediate virulence) and (3) mouse adapted H1N1 A/Puerto Rico/8/34 (PR8; high virulence). Using these mouse models, we demonstrated that rate of viral clearance and disease severity is correlated with the numbers of a defined Gr1 + CD11b + myeloid population in the lung. Until now, it is not clear how and why they accumulate in the severely inflamed lung. In this study, we selected this cell population as a target to investigate the extreme inflammatory response during severe IAV infection.

In this paper, we report that CCR2+ inflammatory monocytes are the major cell components in this defined Gr1 + CD11b + myeloid population. Multiple roles of CCR2+ inflammatory monocytes during viral infections have been reported: promoting host survival of West Nile virus-induced encephalitis and IAV and mouse hepatitis virus infections [9-12], stimulating anti-viral Th1 immunity in HSV-2 infection [13] and suppressing anti-viral CD8 T cell responses in mouse cytomegalovirus (MCMV) and persistent lymphocytic choriomeningitis virus (LCMV) infections [14,15]. These results indicate that CCR2+ inflammatory monocytes play a double edge sword in anti-viral responses and immunopathogenesis. Using established infection models with variable rates of viral clearance, which are accompanied by different levels of inflammatory infiltrates, we found that an amplified inflammatory chemokine feedback loop links the impaired clearance of highly pathogenic virus and a massive infiltration of CCR2+ inflammatory monocytes. So, we sought to investigate which cell types are responsible for the production of CCR2 ligands. Furthermore, we identified the inflammatory signals that are triggered by an impaired anti-viral response to induce expression of CCR2 ligands. Finally, the pathological effects of excessive accumulated CCR2+ inflammatory monocytes were explored during highly pathogenic IAV infection.

Overall, we provided a comprehensive study to address the detail mechanism why and how accumulated CCR2+ inflammatory monocytes involved in highly pathogenic IAV infections. Impaired clearance of virus led to spread of virus to newly arrived CCR2+ inflammatory monocytes and to sustain production of IFNAR1-induced CCR2 ligands, which attract BM-derived CCR2+ monocytes migrated to inflamed lung and amplify their own recruitment continuously through the IFNAR1-dependent chemokine feedback loop, resulting in an enhancement of CCR2+ inflammatory monocytes-mediated pathological effects.

## Methods

### Mouse strains

C57BL/6 and *CCR2*−/− mice were purchased from Jackson Laboratory (Bar Harbor, ME, USA). *IFNAR1*−/− mice were obtained from Dr. Chien-Kuo Lee (Graduate Institute of Immunology, National Taiwan University, Taipei, Taiwan). *MyD88*−/− mice were obtained from Hui-Chen Chen (Graduate Institute of Basic Medical Science, China Medical University, Taichung, Taiwan). Mice were maintained under specific pathogen free conditions in Chang Gung University. All animal experiments were performed according to the animal protocol approved by the Institutional Animal Care and User Committee of Chang Gung University and in accordance with the guidelines of Animal Care and Use of Laboratory Animals of the Taiwanese Council of Agriculture.

### Virus preparation and inoculation

All segmented expression plasmids of IAV were kindly provided by Dr. Shin-Ru Shih of Research Center for Emerging Viral Infections, College of Medicine, Chang Gung University, Taiwan. Recombinant IAVs, seasonal 141, pandemic SOIV and mouse adapted PR8 were generated using a reverse genetics system, according to previous reports [16,17]. Briefly, 293 T cells were transfected by using 15 μl Trans IT-LT1 (Mirus Bio LLC) with 1 μg per each plasmid (pPolI-PB2, −PB1, −PA, −HA, −NP, −NA, −M, −NS of 141, SOIV or PR8). Recombinant IAVs were harvested and propagated in 10 day-old embryonated chicken eggs. Harvested viruses were aliquoted and stored at −80°C until use. For IAV inoculation, mouse was infected intranasally with 200 PFU of virus.

### Plaque assays

Lungs were harvested and grind tissue suspension were frozen in 600 μl aliquots. Viral supernatant was thawed and then 10 folds serially diluted. MDCK cells were cultured at a density of 1 × 10^6^ cells/well in a 6 well-plate. One hundred microliter of each serial dilution containing trypsin was added to 90% confluent of MDCK cells. After 1 hour incubation, each well was overlaid with a ratio of 1:1 mixture of 0.8% agarose and 2× serum free DMEM to wells. Two days later, the plaques were visualized by addition of 1% crystal violet and plaque forming unit (units/lung) was calculated.

### Preparation of lung leucocytes, mediastinal lymph node (MLN), bone marrow (BM) cells and PBMC

Harvested lungs were homogenized using a metal mesh and the suspension was treated with type I collagenase (Invitrogen) per lung for 30 mins at 37°C. Cells were recovered and washed once with complete PRMI medium containing 10% FBS, 1 mM glutamine, 100 U/ml of penicillin and 100 μg/ml of streptomycin. The pelvic and femoral bones were harvested and BM cells were flushed out with complete RPMI medium by insertion of a 1 ml syringe with a 25G needle into one end of the bone. MLN cells were homogenized using glass slides with ground edges. Leukocytes were obtained from the lungs, peripheral blood, BM and MLN after RBC lysis buffer treatment.

### Cytokine antibody array and ELISA

To obtain bronchoalveolar lavage fluid (BALF), airways were flushed three times with 0.5 ml sterile PBS and centrifuged to remove infiltrating cells. Pooled BALFs were assayed using the R & D mouse cytokine arrays (R & D Systems, Inc.) according to the manufacturer’s instructions. CCL2, CCL7 and CCL12 proteins were measured in serum using ELISA kits (eBioscience) according to the manufacturer’s instructions.

### Immunofluorescent surface and intracellular staining

Two million cells were stained with fluorescently labeled mAbs, including Gr1, CD11b, Ly6C, Ly6G, CCR2 and CX3CR1 for 30 min at 4°C. All Abs were purchased from BD Biosciences, except for CCR2 mAb (R & D Systems). After staining, the cells were fixed with Cytofix (BD Biosciences) for 5 min at 4°C. For intracellular staining, cells were stained with fluorescently labeled anti-Gr1, −CD11b and -Ly6C mAbs and then fixed with Cytofix/cytoperm (BD Biosciences) for 20 mins at 4°C. Fixed cells were further stained with FITC-labeled anti-IAV nucleoprotein (NP) Ab (Abcam) for another 30 min at 4°C. Finally, the cells were washed and re-suspended in FACS buffer (PBS with 2% FBS) and analyzed by LSRII flow cytometry (BD Biosciences).

### Cell sorting and Wright stain

Infiltrating leukocytes from the lungs were harvested and incubated with anti-Gr1, −CD11b and -Ly6G mAbs. Gr1 + CD11b+, Gr1-CD11b-, Gr1 + CD11b + Ly6G + (for granulocyte sorting) or Gr1 + CD11b + Ly6G- (for monocyte sorting) leukocytes were sorted by FACS Aria (BD Biosciences). For morphological evaluation of Gr1 + CD11b + cells, sorted cells were spun onto glass slides at 250 rpm for 5 min using a Shandon Cytospin 3 Centrifuge (Global Medical Instrumentation Inc.) and stained with HemaTek stain Pak (Siemens Healthcare Diagnostic Inc.) using an automatic HemaTek hematology stainer (Bayer Healthcare, LLC.).

### RNA extraction, reverse transcription and quantitative polymerase chain reaction (RT-QPCR)

Total RNA was extracted from isolated or sorted cells using TRIzol reagent (Invitrogen) according to the manufacturer’s instructions. RNA was used to synthesize cDNA with Superscript III reverese transcriptase (Invitrogen). TaqMan® Gene Expression Assays (Applied Biosystems) were performed to detect mouse CCL2, CCL7, CCL12, iNOS, IFNβ and GADPH mRNAs. Expression of the various genes was normalized with the GADPH level in each group. Relative gene expression was determined using △△Ct analysis.

### Western blotting

Frozen lung tissues were lysed using lysis buffer (100 mM Tris, 250 mM NaCl, 0.5% sodium deoxycholate, 1 mM PMSF and 0.5% NP40). Tissue lysates were resolved by electrophoresis in SDS-polyacrylamide gels and electrotransferred onto Hybond-P PVDF membranes (GE Healthcare). Milk blocked blots were incubated with anti-actin and -NP antibodies at 4°C overnight and then washed and incubated with horseradish peroxidase (HRP)-conjugated secondary antibodies (Jackson ImmunoResearch) at room temperature for 1 hr. The proteins were revealed using the Immobilon Western Chemiluminescent HRP Substrate (Millipore).

### Oseltamivir treatment

PR8-infected mouse was treated with 50 mg Oseltamivir daily according to a previous report [18].

### Treatment with anti-IFNAR1 blocking antibody

Day 3 post-infected mice were anesthetized and then injected intranasally with either 50 μg of IgG isotype control antibody (Abcam) or 50 μg of anti-IFNAR1 antibody (eBioscience). After 3 days, infiltrating cells were counted and then stained with specific Abs against with Gr1, CD11b, Ly6G, Ly6C and CCR2.

### Adoptive transfer of BM enriched CCR2+ monocytes into mice

BM cells from naïve B6 mice were harvested and monocytes were enriched by negative selection using an EasySep™ Mouse Monocyte Enrichment Kit and EasySep™ magnet system (STEMCELL Technologies Inc.). Enriched monocytes were suspended in PBS at a concentration of 2.0 × 10^7^ cells/ml and incubated with 5 μM carboxyfluorescein diacetate succinimidyl ester (CFSE, Invitrogen) solution for 12 min at 37°C. One million CFSE-labeled cells were adoptively transferred via the tail vein into naïve or virus-infected mice. After 2 days, leukocytes were harvested from the lungs and stained with anti-Ly6C and anti-CCR2 antibodies. Finally, CCR2 + CFSE + transferred monocytes were traced using flow cytometric analysis.

### Statistical analysis

Statistical significance of the data was analyzed by Student’s two-tailed t test.

## Results

### Excessive accumulation of CCR2+ inflammatory monocytes in severe IAV infection

**Figure 1:**
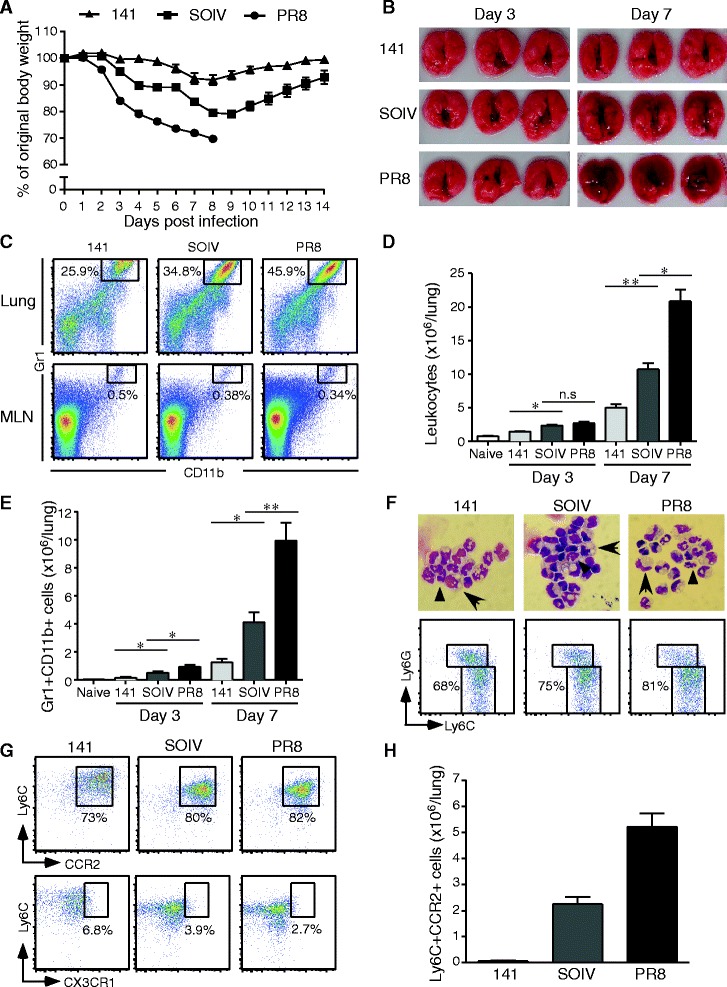
**Excessive accumulation of CCR2+ inflammatory monocytes in severe IAV infection.** C57BL/6 mice were infected with 200 PFU of 141, SOIV or PR8 viruses. **(A)** Body weights were monitored daily until day 14 post-infection (n = 6 -8 per group, mean ± SEM). **(B)** Appearance of lung inflammation was photographed at days 3 and 7 post-infection (n = 3 per group). **(C)** Total leukocytes were stained with Abs against Gr1 and CD11b. The percentage of Gr1 + CD11b + myeloid cells was analyzed by flow cytometry. **(D)** Total leukocytes were harvested from the lungs at the time points indicated and counted by trypan blue exclusion. These data are a composite of four to seven independent experiments (n = 3 per group, mean ± SEM; n.s: no significant difference; *P < 0.05; **P < 0.01). **(E)** Numbers of Gr1 + CD11b + myeloid cell of lung were shown. These data are a composite of four independent experiments (n = 3 per group, mean ± SEM; ns: no significant difference; *P < 0.05; ** P < 0.01). (**F**, upper panel) Gr1 + CD11b + cells were sorted from infiltrating leukocytes and then stained by Wright stain. The cell morphology was photographed under 1000× magnification using an Olympus microscope. Granulocytes are indicated by arrow heads and monocytes are indicated by arrows. (F, lower panel). The percentage of Ly6G-Ly6C^high^ monocytes in the Gr1 + CD11b + gated population is shown. Dot plots are the representative result from three repeated experiments with three mice per group. **(G)** The percentage of CCR2+ inflammatory monocytes and CX3CR1 patrolling monocytes in Gr1 + CD11b + myeloid cells. **(H)** Numbers of Ly6C^high^CCR2+ inflammatory monocytes were shown at day 7 post-infection. This is a representative result from four repeated experiments with three mice per group.

We observed varying levels of body weight change and lung inflammation in the infected mice and investigated which infiltrating cell type was associated with severe inflammation. As shown in Figure 1A, mice infected with the mild 141 strain lost 5%-10% of their original body weight, while the moderate SOIV strain caused 15%-20% original body weight loss. Notably, severe PR8 infection caused progressive weight loss and led to 100% mortality in the infected mice at day 7–10 post-infection. In these infections, lung inflammation was dramatically correlated with body weight loss at day 7 post-infection (Figure 1B). Furthermore, we demonstrated that a defined Gr1 + CD11b + myeloid population is preferentially recruited to the infected lung, but only few to MLN (Figure 1C). Of interest, the total numbers of infiltrating leukocytes and Gr1 + CD11b + cells were significantly associated with the severity of inflammation (Figure 1D and E). Gr1 + CD11b + cells are a heterogeneous cell population, so the true identity of major infiltrating cells should be further characterized using the Wright stain and by the expression of Ly6G and Ly6C on cell surface. Gr1 + CD11b + sorted cells consisted mostly of mononuclear cells containing abundant cytoplasmic vacuoles and few segmented granulocytes (Figure 1F, upper panel). Furthermore, Gr1 + CD11b + cells are composed of appoximately 68-81% monocytes (Ly6G-Ly6C^high^) and 19-32% granulocytes (Ly6G + Ly6C^intermediate^) (Figure 1F, lower panel). Using specific Abs against surface CCR2 and CX3CR1, we further demonstrated that the infiltrating monocytes in the lungs were Ly6C^high^CCR2+ inflammatory monocytes but not Ly6C^low^CX3CR1+ patrolling monocytes (Figure 1G). Importantly, the numbers of infiltrating CCR2+ inflammatory monocytes were highly associated with the severity of inflammation (Figure 1H).

### Cytokine and chemokine profiling of BALFs

**Figure 2:**
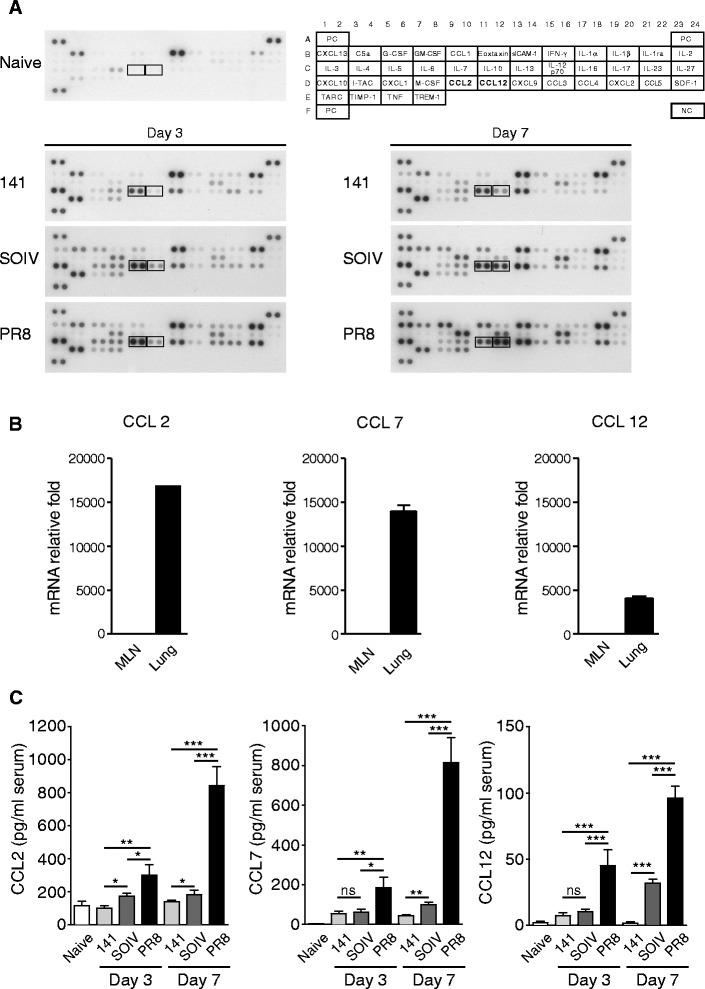
**Expression of CCR2 ligands in BALF, MLN, lung and serum of infected mice.** C57BL/6 mice were infected with 200 PFU of 141, SOIV or PR8 viruses. BALF was harvested from naïve or virus-infected mice at days 3 and 7 post-infection (n = 3–4 mice per group). **(A)** Pooled BALFs were subjected to cytokine or ckemokine expression analysis using cytokine protein arrays. **(B)** Mice were infected with PR8 viruses. At day 7 postinfection, RNAs were harvested from isolated MLN and lung leukocytes. Relative expression of CCL2, CCL7 and CCL12 in total leukocytes from MLN and lung was measured by RT-QPCR. The mRNA relative folds were determined by normalizing the level of each group to the corresponding GAPDH level and then to total leukocytes from MLN (mean ± SEM). Experiment (n = 3 mice per group) was performed twice and one representative is shown. **(C)** Sera were collected from naïve and virus-infected mice at days 3 and 7 post-infection. Concentrations of CCL2, CCL7 and CCL12 in the sera were measured by ELISA (n = 6–11 mice per group, mean ± SEM; ns: no significant difference; *P < 0.05; **P < 0.01; ***P < 0.001).

To investigate the mechanism of extensive accumulation of CCR2+ inflammatory monocytes in severe inflammation, the cytokines and chemokines listed in Figure 2A were evaluated. According to the results of protein arrays, levels of G-CSF, CCL1, CCL2, CCL12, IL-10, CXCL9, IL-16 and CCL5 were correlated with the severity of lung inflammation (Figure 2A). Notably, both CCL2 and CCL12 are ligands of CCR2, in addition to CCL7. So, we speculated that the aggressive recruitment of CCR2+ inflammatory monocytes is linked to expression of CCR2 ligands. In Figure 1C, we found that Gr1 + CD11b + cells preferentially migrate to the lung but not to MLN. Therefore, we suggested that leukocytes infiltrating the lung may frequently induce CCR2 ligands to attract CCR2+ inflammatory monocytes. Indeed, all transcripts of CCR2 ligands were over 4000 fold higher in the lung than in MLN (Figure 2B). In addition, the levels of CCR2 ligands in sera were clearly correlated with the numbers of infiltrating CCR2+ inflammatory monocytes (Figure 2C). These results suggested that robust expression of CCR2 ligands may contribute to the aggressive recruitment of CCR2+ inflammatory monocytes into the lungs.

### Induction of CCR2 ligands by CCR2+ inflammatory monocytes

**Figure 3:**
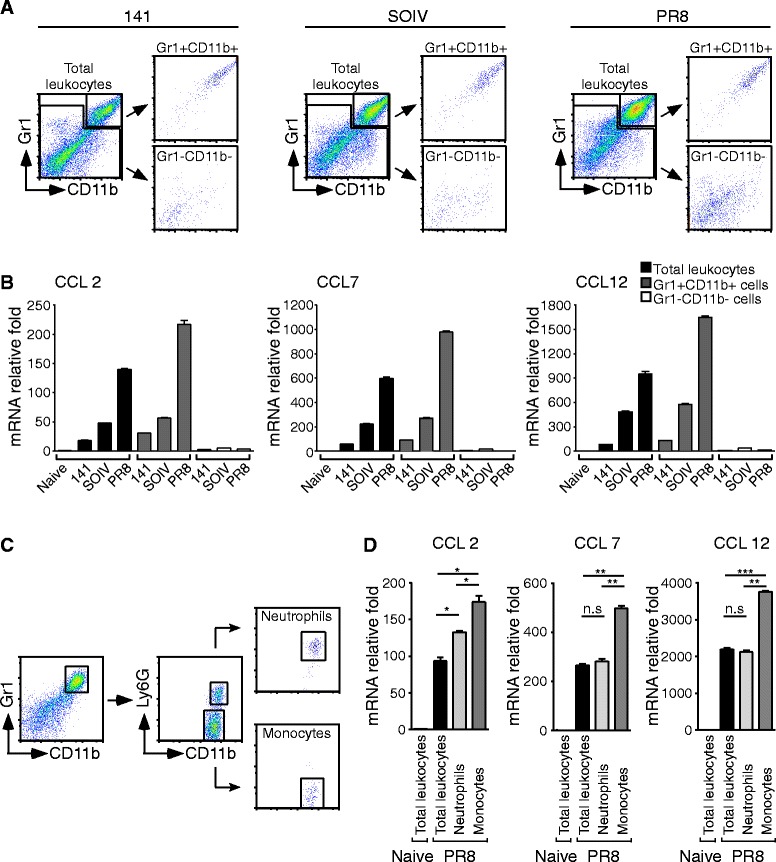
**CCR2+ inflammatory monocytes produce high amounts of CCR2 ligand.** Total leukocytes were harvested from naïve or virus-infected mice at day 7 post-infection. **(A)** Gr1 + CD11b + and Gr1-CD11b- cells were sorted form total leukocytes using a cell sorter. **(B)** RNAs were extracted from total leukocytes, Gr1 + CD11b + sorted cells and Gr1-CD11b- sorted cells from the virus-infected mice indicated. Experiment (n = 3-6 mice per group) was performed at least twice and one representative is shown. **(C)** Isolated leukocytes were stained with anti-Gr1, −CD11b and –Ly6G Abs. Gr1 + CD11b + Ly6G + and Gr1 + CD11b + Ly6G- cells were sorted. **(D)** RNA was extracted from total leukocytes, Gr1 + CD11b + Ly6G + sorted cells and Gr1 + CD11b + Ly6G- sorted cells from PR8-infected mice and expression of CCL2, CCL7 and CCL12 was measured by RT-QPCR. The mRNA relative folds were determined by normalizing the level of each group to the corresponding GAPDH level and then to total leukocytes from naïve mice (mean ± SEM; ns: no significant difference; *P < 0.05; **P < 0.01; ***P < 0.001). Experiment (n = 4–5 mice per group) was performed twice and one representative is shown.

We sought to determine whether the infiltrating Gr1 + CD11b + cells are possible producers of CCR2 ligands. To test this possibility, total infiltrating leukocytes were separated into Gr1 + CD11b + cells and Gr1-CD11b- cells using a cell sorter (Figure 3A). Compared to leukocytes in the lungs of naïve mice, infiltrating leukocytes harvested from virus-infected mice had tens- to thousands-fold induction of CCR2 ligands (Figure 3B). The relative fold induction of CCR2 ligands was similar between total leukocytes and Gr1 + CD11b + sorted cells, suggesting that Gr1 + CD11b + cells are probably the main producers of CCR2 ligands. To confirm that CCR2+ inflammatory monocytes were producers of CCR2 ligands, granulocytes and monocytes were sorted from the Gr1 + CD11b + myeloid population (Figure 3C). As shown in Figure 3D, both cell types could express CCL2, CCL7 and CCL12, but more expression of these CCR2 ligands was seen in monocytes. Thus, our results suggested that infiltrating CCR2+ inflammatory monocytes act positively in a chemokine feedback loop to recruit more CCR2+ inflammatory monocytes.

### Induction of CCR2 ligands is dependent on IFNAR1-triggered signaling

**Figure 4:**
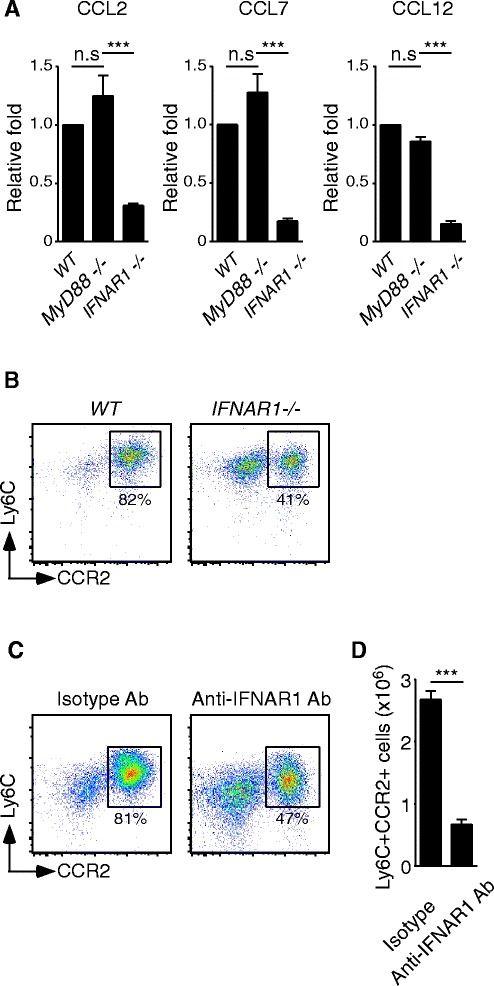
**Induction of CCR2 ligands is dependent on the IFNAR1-triggered signaling. (A)** Gr1 + CD11b + myeloid cells were isolated from the lungs of PR8-infected WT, *MyD88*−/− and *IFNAR1*−/− mice and RNAs were extracted. Expression of CCL2, CCL7 and CCL12 was measured by RT-QPCR. The mRNA relative folds were determined by normalizing the level of each group to its GAPDH and then to WT infected mice (mean ± SEM; ns: no significant difference; ***P < 0.001). These data are a composite of three independent experiments (n = 6 mice per group). **(B)** Total leukocytes were isolated from PR8-infected WT, *MyD88−/−* or *IFNAR1*−/− mice and the cells were stained with anti-Gr1, −CD11b, −Ly6C and -CCR2 Abs. The percentage of Ly6C^high^CCR2+ inflammatory monocytes in Gr1 + CD11b + gated cells is shown. This is a representative result of two repeated experiments with two-three mice per group. **(C-D)** Day 3 PR8-infected mice were treated either with isotype control antibody or anti-IFNAR1 blocking antibody. After 3 days, leukocytes were harvested and stained with anti-Gr1, −CD11b, −Ly6C and -CCR2 Abs. The percentage of Ly6C^high^CCR2+ inflammatory monocytes in Gr1 + CD11b + gated cells and numbers of Ly6C^high^CCR2+ inflammatory monocytes are shown (mean ± SEM; ***P < 0.001). These data are a composite of two independent experiments (Isotype control, n = 5; anti-IFNAR1 Ab treatment, n = 6).

We next sought to determine which inflammatory signaling pathway was responsible for the induction of CCR2 ligands. Previous studies indicated that signaling pathways of MyD88 and type I IFN could modulate the recruitment of myeloid cells [19,20]. Therefore, *MyD88−/−* and *IFNAR1−/−* mice were used in this study. Compared to infected WT and *MyD88*−/− mice, the expression of CCR2 ligands by Gr1 + CD11b + cells was significantly reduced in infected *IFNAR1*−/− mice (Figure 4A). In addition, we found that the percentage of CCR2+ inflammatory monocytes was only reduced in *IFNAR1*−/− mice. In Figure 4B, CCR2+ inflammatory monocytes accounted for 81.8 ± 1.1% of total leukocytes in infected WT mice and 84.5 ± 4.5% in infected *MyD88* deficient mice; however, CCR2+ inflammatory monocytes accounted for only 39.8 ± 0.35% in infected *IFNAR1*−/− mice. Thus, accumulation of CCR2+ inflammatory monocytes was suppressed when the IFNAR1-induced expression of CCR2 ligands was interrupted. Because aggressive recruitment of Gr1 + CD11b + cells was observed after day 3 post-infection (Figure 1E), we wondered whether intranasal treatment with an anti-IFNAR1 blocking antibody at day 3 post-infection could interrupt the influx of CCR2+ inflammatory monocytes. In Figure 4C and D, the recruitment of CCR2+ inflammatory monocytes was reduced significantly in anti-IFNAR1 blocking antibody-treated mice, but not in isotype control-treated mice. Overall, these data implied that excessive recruitment of CCR2+ inflammatory monocytes contributes to continuous activation of IFNAR1-induced expression of CCR2 ligands.

### Impaired anti-viral responses prolong IFNβ expression

**Figure 5:**
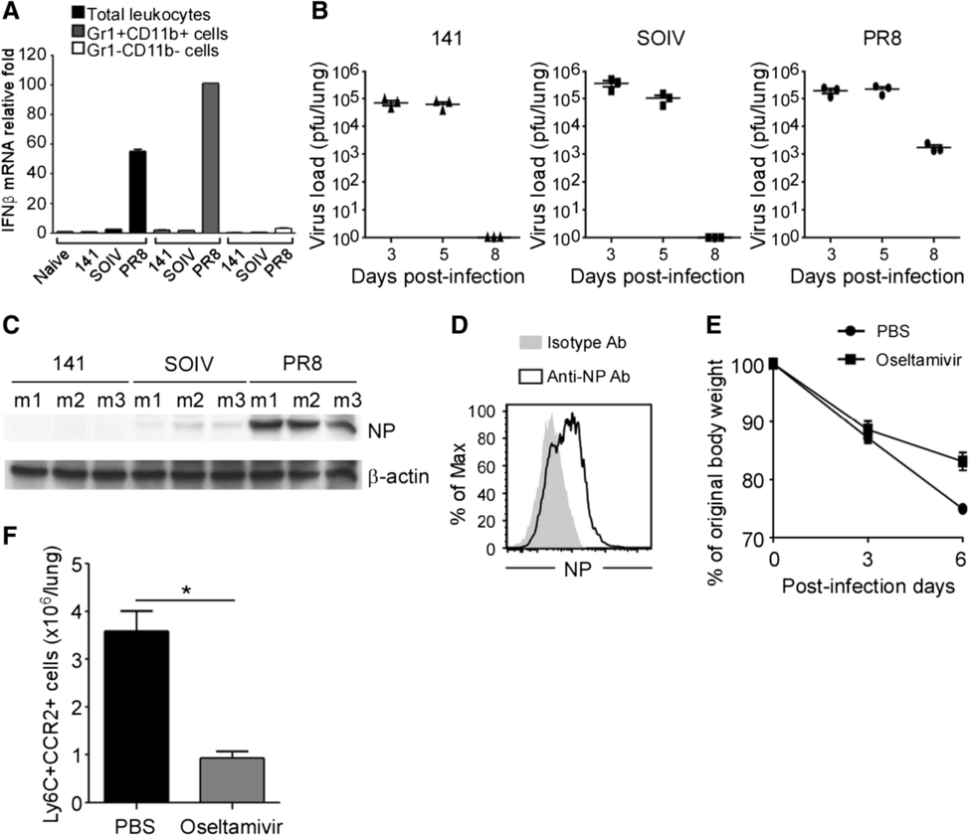
**Impaired clearance of viral replication sustains IFNβ production.** Total leukocytes were harvested from naïve or virus-infected mice at day 7 post-infection. **(A)** RNA was extracted from total leukocytes, Gr1 + CD11b + sorted cells and Gr1-CD11b- sorted cells. Expression of IFNβ was measured by RT-QPCR. The mRNA relative folds were determined by normalizing the level of each group to the corresponding GAPDH level and then to total leukocytes from naïve mice (mean ± SEM). Experiment (n = 3–6 mice per group) was performed twice and one representative is shown. **(B)** Lungs were harvested from virus infected mice at the time points indicated and the virus load was measured by plaque assays (n = 3 mice per group; mean ± SEM). **(C)** Protein lysates of lungs were harvested from infected mice (n = 3 mice per group) on day 7 and expression of influenza NP was detected by western blotting; β-actin expression served as the internal control. This is a representative result from three repeated experiments. **(D)** Total leukocytes were harvested from PR8-infected mice at day 7 post-infection. Expression of influenza NP in Ly6C^high^CCR2+ cells was detected by flow cytometry. This is a representative result from three repeated experiments. **(E)** Body weight changes of PBS- and Oseltamivir-treated mice were monitored at day 0, 3 and 6 post-infection (mean ± SEM). **(F)** Leukocytes were harvested from PBS- and Oseltamivir-treated mice at day 6 post-infection. Cells were stained with Abs against Gr1, CD11b, Ly6C and CCR2, and then CCR2+ monocytes were analyzed by flow cytometry. The numbers of CCR2+ inflammatory monocytes were calculated in each group. These data are a composite of two independent experiments (n = 4 mice per group, mean ± SEM; *P < 0.05).

Type I IFNs (IFNα and IFNβ) are considerd to bind the heterodimeric complexes of IFNAR1 and IFNAR2. Recent study has shown that induction of CCL2 and CCL7 is triggered by the IFNAR1-IFNβ signaling in *IFNAR2−/−* mice [21]. In addition, we also observed differential expression of CCR2 ligands among Gr1 + CD11b + sorted cells in 141, SOIV and PR8 infections (Figure 3B). Therefore, we examined the expression levels of IFNβ in all infected mice. As expected, expression of IFNβ as detected only in the Gr1 + CD11b + sorted cells harvested from PR8-infected mice at day 7 post-infection (Figure 5A). In addtion, both granulocytes and monocytes in Gr1 + CD11b + population could express IFNβ (data not shown). Because detectable IFNβ production reflects activated viral replication, the anti-viral responses of the host were examined by measuring virus titers and detecting influenza NP expression in the infected lung. As shown in Figure 5B and C, 141-infected mice completely eliminated the virus at day 7. SOIV-infected mice still showed weak expression of NP at day 7 and the host completely cleared the virus at day 8 post-infection. Of note, PR8-infected lungs still showed strong NP expression and viral replication at day 7–8 post-infection. These data suggested that the duration of IFNβ production is a function of the rate of viral clearance. Next, we sought to explore why Gr1 + CD11b + cells produce abundant IFNβ in PR8-infected mice in the late phase of infection. We hypothesized that recruited CCR2+ inflammatory monocytes are infected by the PR8 virus, resulting in amplified production of IFNβ. Indeed, expression of influenza NP was detected in CCR2+ inflammatory monocytes in PR8-infected mice (Figure 5D). Thus, our results suggested that impaired clearance of PR8 virus prolonged expression of IFNβ, which led to infected CCR2+ inflammatory monocytes amplifying their own recruitment by an IFNAR1-triggered chemokine feedback loop. To determine whether high viral loads are potent inducers for CCR2+ monocyte infiltration, an anti-viral drug, Oseltamivir, was used to suppress virus replication in infected mice. In Figure 5E, body weight loss was attenuated when infected mice received Oseltamivir treatment, demonstrating the efficacy of Oseltamivir. Influx of CCR2+ inflammatory monocytes was dramatically reduced in Oseltamivir-treated mice, compared to PBS-treated mice (Figure 5F). Taken together, our results supported the concept that continuous recruitment of CCR2+ inflammatory monocytes by the IFNAR1-triggered chemokine feedback loop is attributable to the extended duration of IFNβ expression in the late phase of infection.

### The balance of CCR2+ inflammatory monocytes between the BM and lungs

**Figure 6:**
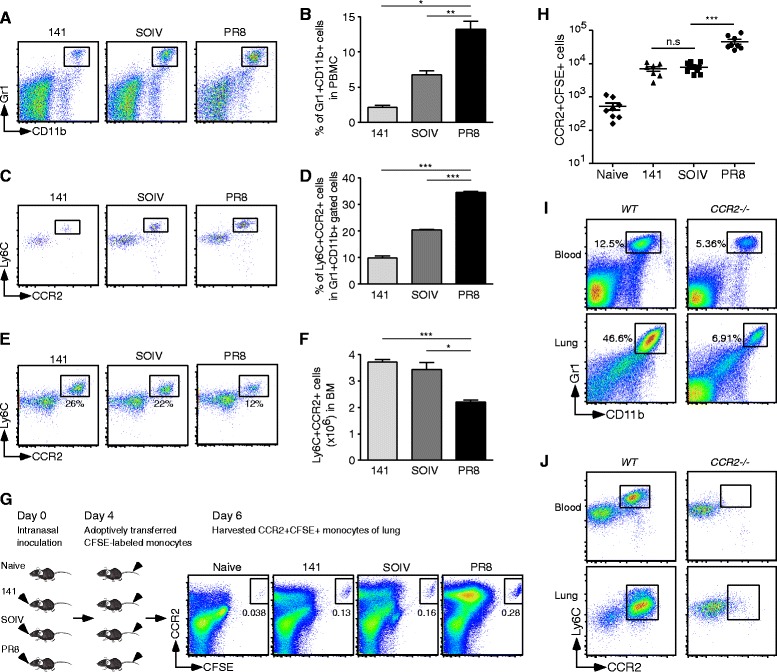
**Balance of CCR2+ inflammatory monocytes between the BM and lung. (A-F)** PBMC and BM cells were harvested from infected mice at day 7 post-infection. **(A-B)** The dot plots and percentage of Gr1 + CD11b + cells in PBMC are shown. This data are a composite of three independent experiments (n = 9 mice per group, mean ± SEM, *P < 0.05; ** P < 0.01). **(C-D)** The dot plots and percentage of Ly6C^high^CCR2+ monocytes in the Gr1 + CD11b + gated cells in PBMC are shown. This data are a composite of two independent experiments (n = 3 mice per group, mean ± SEM, ***P < 0.001). **(E-F)** The dot plots and numbers of CCR2+ monocytes were counted from indicated virus infections at day 7 post-infection. These data are a composite of four independent experiments (mean ± SEM; *P < 0.05; ***P < 0.001). **(G-H)** CFSE-labeled monocytes of BM were transferred into naïve or day 4 post-infected mice. After 2 days, infiltrating leukocytes were harvested from the lungs and stained with a specific Ab against CCR2. **(G)** The percentage of CCR2 + CFSE + transferred cells is shown for the groups indicated. **(H)** Absolute numbers of transferred CCR2 + CFSE + monocytes in the lungs were counted in each group. These data are a composite of three independent experiments (n = 7 to 8 mice per group, means ± SEM; ns: no significant difference; ***P < 0.001). **(I)** Percentage of Gr1 + CD11b + cells in PBMC or lung is shown. **(J)** Ly6C^high^CCR2+ inflammatory monocytes in Gr1 + CD11b + gated population in PBMC or lung are shown. This is a representative result from three repeated experiments.

We next examined the source of infiltrating CCR2+ inflammatory monocytes in the host. In general, CCR2+ inflammatory monocytes are generated from the BM and migrate rapidly to inflamed sites following pathogen invasion [22]. Therefore, we first checked the proportions of Gr1 + CD11b + cells and CCR2+ inflammatory monocytes in the PBMC and BM during infection. As shown in Figure 6A-D, the proportion of Gr1 + CD11b + cells and CCR2+ inflammatory monocytes in the PBMC was positively correlated with disease severity. In contrast, the proportion of CCR2+ monocytes in the BM was inversely correlated with the severity of inflammation (Figure 6E). In Figure 6F, total CCR2+ monocytes were significantly decreased in the BM of PR8-infected mice, compared to those in 141- and SOIV-infected mice. These results implied that CCR2+ monocytes are rapidly recruited from the BM to the infected lung and mobilization of these cells is possibly dependent on the expression of CCR2 ligands. To demonstrate CCR2-mediated trafficking of inflammatory monocytes to the lungs, BM-enriched CCR2+ monocytes were isolated from naïve mice, labeled with CFSE, and then adoptively transferred to naïve, 141-, SOIV- or PR8-infected mice. After 2 days, transferred CCR2+ inflammatory monocytes were traced by the CCR2 + CFSE + signals on the cells (Figure 6G). In Figure 6H, more transferred CCR2 + CFSE + monocytes were found in PR8-infected lungs than in those 141- and SOIV-infected lungs. *CCR2*−/− mice were used to confirm that influx of CCR2+ inflammatory monocytes was dependent on CCR2-triggered chemotaxis. In Figure 6I, the proportion of Gr1 + CD11b + cells was significantly decreased in infected *CCR2*−/− mice, compared to WT mice. In Figure 6J, only few CCR2+ inflammatory monocytes were detected in the blood and lungs of infected *CCR2*−/− mice, suggesting the importance of CCR2-driven monocytes localization within the infected lung.

### Pathological effects of CCR2+ inflammatory monocytes upon IAV infection

**Figure 7:**
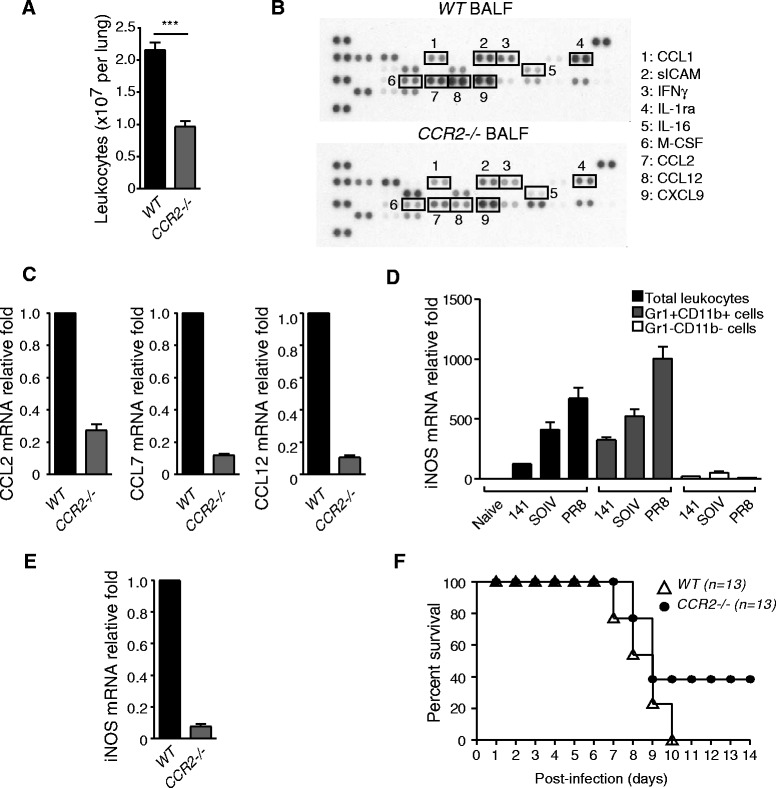
**Decreasing pathological effects in PR8-infected** ***CCR2*** **−/− mice. (A)** Total leukocytes were harvested from the lungs and counted by trypan blue exclusion. These data are a composite of three independent experiments (n = 9 mice per group, mean ± SEM; ***P < 0.001). **(B)** Pooled BALFs were subjected to cytokine or chemokine expression analysis using cytokine protein arrays (n = 6 mice per group). **(C)** Relative expression of CCL2, CCL7 and CCL12 was measured by RT-QPCR. The mRNA relative folds were determined by normalizing the level of each group to the corresponding GADPH level and then to total leukocytes from WT mice (mean ± SEM). This is a representative result from two repeated experiments. **(D)** RNAs were extracted from total leukocytes, Gr1+ CD11b+ sorted cells and Gr1-CD11b- sorted cells from the virus-infected mice indicated. Relative expression of iNOS transcripts was measured by RT-QPCR. The mRNA relative folds were determined by normalizing the level of each group to the corresponding GAPDH level and then to total leukocytes from naïve mice (mean ± SEM). Experiment (n = 3–6 mice per group) was performed twice and one representative is shown. **(E)** RNAs were harvested from leukocytes isolated from the lungs of WT and *CCR2*−/− infected mice. Relative expression of iNOS was measured by RT-QPCR. The mRNA relative folds were determined by normalizing the level of each group to the corresponding GAPDH level and then to total leukocytes from WT mice (n = 3 mice per group; mean ± SEM). Experiment was performed twice and one representative is shown. **(F)** WT (n = 13) and *CCR2*−/− mice (n = 13) were infected with PR8 viruses. Survival rate was monitored daily until day 14 post-infection. These data are a composite of three independent experiments.

A previous study showed that monocytes are retained in the BM when they lack CCR2 expression [23]. To investigate the biological consequences of an excessive accumulation of CCR2+ inflammatory monocytes in the lungs, CCR2−/− mice were used to examine leukocyte infiltration, cytokine storm, expression of iNOS and the survival rate after a lethal dose challenge of PR8 virus. In the absence of infiltrating CCR2+ inflammatory monocytes, total leukocytes in the lung and expression of CCL1, sICAM-1, IFNγ, IL-1ra, IL-16, M-CSF, CCL2, CCL12 and CXCL9 in BALF were decreased, suggesting that CCR2+ inflammatory monocytes contribute to the expression of these molecules (Figure 7A and B). Consistent with the results from cytokine arrays, expression of CCR2 ligands was also significantly decreased in the infiltrating leukocytes of *CCR2*−/− mice, compared to WT mice (Figure 7C). A previous report has shown that iNOS is induced in activated myeloid cells and significantly involved in the development of IAV-induced pneumonitis [24]. As shown in Figure 7D, Gr1 + CD11b + cells were the predominant producers of iNOS. Interestingly, expression of iNOS was correlated with the severity of inflammation. To demonstrate further the importance of CCR2+ inflammatory monocytes-mediated immunopathological effects, expression of iNOS and the survival rate were compared in PR8-infected WT and *CCR2*−/− mice. Expression of iNOS transcripts was dramatically reduced in infected *CCR2*−/− mice, (Figure 7E). Finally, 38.5% of infected *CCR2*−/− mice, but none of the WT mice, survived a lethal dose challenge of PR8 virus (Figure 7F). Thus, infiltrating CCR2+ inflammatory monocytes play a pivotal role in highly virulent IAV infection-mediated pathological effects.

## Discussion

IAV not only infect pulmonary epithelial cells, endothelial cells and resident alveolar macrophages but also infiltrating granulocytes, monocytes and dendritic cells [18,25]. Furthermore, infected leukocytes are the main contributors to aggressive production of inflammatory innate immune responses. Before entering the inflamed lung, these uninfected infiltrates are already primed with type I IFN, which upregulates the levels of MAD5, RIG-I and IRF7 [26,27]. In addition, these IFN-stimulated molecules coupled with viral nucleic acids are responsible for amplified production of type I IFN [28]. Our findings revealed that the rate of virus clearance determines the duration of IFNβ expression in infiltrating Gr1 + CD11b + cells. Sustained expression of IFNβ was critical for aggressive recruitment of CCR2+ inflammatory monocytes in severe inflammation. When virus replication was suppressed by Oseltamivir, body weight and influx of CCR2+ inflammatory monocytes were significantly reduced. These results indicated that excessive accumulation of CCR2+ inflammatory monocytes plays a crucial role in the pathological outcomes of highly pathogenic H1N1 IAV infections. Recently, we are continuously threatened by sporadic infections by emerging avian influenza viruses, including highly pathogenic avian H5N1 and H7N9 viruses which rapidly develop acute respiratory distress syndrome, including excessive infiltration of neutrophils and monocytes into the lungs, high viral loads and hypercytokinemia [29,30]. Futhermore, it is worth to see whether the same phenomenon is observed in avain flu infections, such as H5N1 and H7N9. If accumulation of CCR2+ inflammatory monocytes is a common phenomenon in highly pathogenic influenza infection, CCR2+ inflammatory monocytes will be a good therapeutic target in infection.

The mechanism of accumulation of CCR2+ inflammatory monocytes in severe IAV infection remains largely unclear. Previous reports have shown that small numbers of neutrophils are recruited early in infection, followed by influx of large numbers of monocytes [4]. Based on our result, CCR2 ligands produced by neutrophils might play a key role in the early recruitment of monocytes. In Figure 1F, the ratio of neutrophils and monocytes was skewed according to degrees of inflammation at day 7 post-infection. This result indicated two things: (1) Monocyte attractive chemokines were not only provided by neutrophils but also by other inflammatory cells. Using cell sorting, we showed that accumulated CCR2+ inflammatory monocytes are the main contributors of CCR2 ligands and then amplify their own recruitment. (2) Infiltrating monocytes might interfere with the further influx of neutrophils in severe inflammation. A previous study has demonstrated that type I IFN suppresses neutrophil-mediated chemokine attraction, CXCL1 and CXCL2, leading to impaired recruitment of neutrophils [31]. Therefore, we suggested that sustained expression of IFNβ from CCR2 inflammatory monocytes interrupts the recruitment of neutrophils. Indeed, our data are consistent with previous reports that a doubling of neutrophil numbers is observed in *CCR2*−/− and IFNAR1−/− mice [20,32].

Production of type I IFN is a double edged sword in terms of viral clearance and virus-mediated pathogenesis. Previous studies have shown that prolonged induction of type I IFN was seen in highly virulent IAV infections, leading to severe consequences: (1) Type I IFN-induced apoptosis of alveolar epithelial cells by TRAIL is observed in severe IAV infections [33]. (2) Type I IFN-induced FasL expression in the epithelial cells of the lung contributes to the severity of infection [34]. (3) Type I IFN mediates the development of post-influenza bacterial infections [31]. In our study, CCR2+ inflammatory monocytes amplify their own recruitment by a prolonged IFNAR1-triggered chemokine feedback loop.

In our study, induction of CCR2 ligands in CCR2+ inflammatory monocytes was dependent on the IFNAR1-triggered signaling pathway. However, recruitment of CCR2+ inflammatory monocytes could not be completely abolished in *IFNAR1−/−* mice, suggesting that induction of CCR2 ligands in other cell types by IFNAR1-independent pathways may not be excluded. Indeed, expression of CCL2 is regulated by Sphingosine-1-phosphate receptor-triggered signaling in pulmonary endothelial cells or by the MyD88-mediated pathway in pulmonary epithelial cells [35-37]. In addition, IL-1R signaling is also involved in CCL2 induction in undefined cell types during IAV infection [38]. A previous study has demonstrated that mice deficient in a single ligand, either CCL2 or CCL7, only can block 40-50% monocytes egressing from the BM [39]. Thus, it is not surprising that gene deficiency of CCL2 cannot protect mice against highly pathogenic virus-mediated death [7]. Our results indicated that CCL2, CCL7 and CCL12 were highly induced during IAV infections. Therefore, blockage of any single CCR2 ligand is not sufficient to block recruitment of CCR2+ inflammatory monocytes.

Recuirted CCR2+ inflammatory monocytes play a critical role in innate and adaptive immune responses during IAV infections. In successful clearance of 141 and SOIV infecions, CCR2+ inflammatory monocytes expressed high levels of IFNγR, MHC class I and MHC class II molecules than those molecules on monocytes isolated from PR8-infected mice (data not shown). Our and previous studies have demonstrated that monocytes are the mainly susceptible cell type to IAV infections [40,41]. Therefore, we suggested that the rate of viral clearance markedly determines the functional direction of inflitrating CCR2+ inflammatory monocytes toward in either protective or pathological role. In infections of MCMV and LCMV, CCR2+ inflammatory monocyte-produced large amount of iNOS and facilitate the production of nitric oxide (NO). NO plays a critical role to impair anti-CD8 T cell responses [14,15]. In our study, CCR2+ inflammatory monocytes expressed iNOS; and its expression was correlated with rate of viral clearance. Thus, these results implied that excessive accumulation of CCR2+ inflammatoy monocytes might interfere effective anti-viral CD8 T-cell responses via excessive NO prodution in highly pathogenic IAV infections.

In summary, overabundant innate immune responses produced by monocytes contribute significantly to highly pathogenic virus-mediated fatal outcomes. Based on our findings, the proportion of CCR2+ inflammatory monocytes in the blood and concentration of CCR2 ligands in the serum have potential as translational biomarkers to predict IAV virulence and pathogenesis in an emerging pandemic infection and sporadic infections of avian IAVs. In addition, inhibition of recruitment of CCR2+ inflammatory monocytes or depletion of infiltrating CCR2+ inflammatory monocytes may provide an alternative immunotherapeutic way to reduce the damaging effects of accumulating CCR2+ inflammatory monocytes in highly pathogenic IAV infections.

## Conclusion

**Figure 8:**
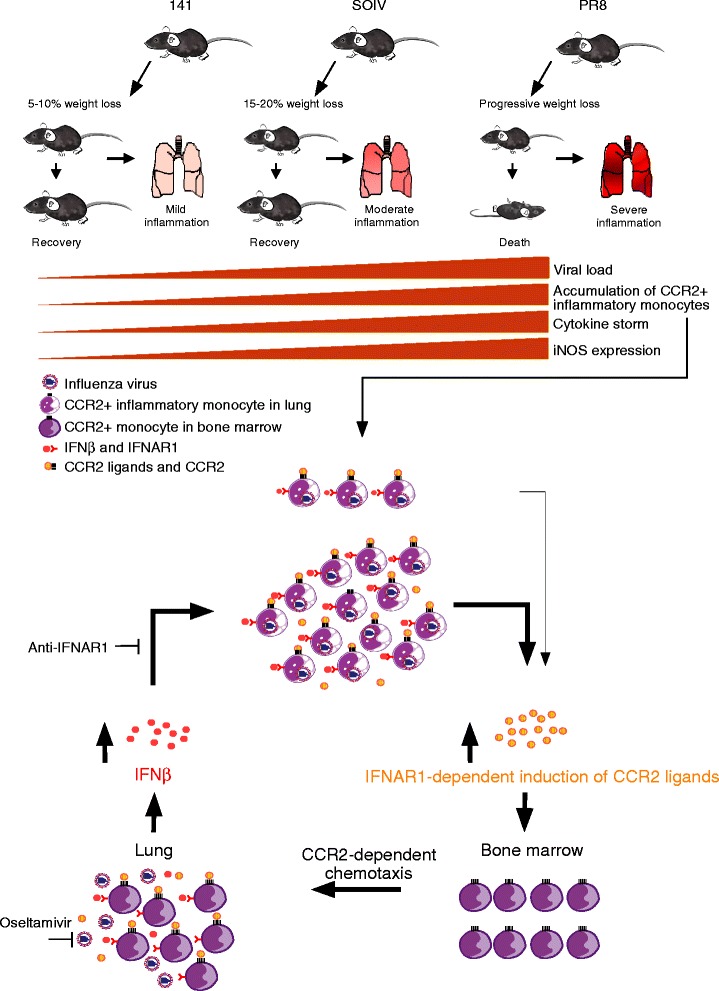
**Roles of CCR2+ inflammatory monocytes in highly pathogenic IAV infection.** We achieved varying degrees of weight loss with mildly, moderately or severely inflamed lungs in mice inoculated with the 141, SOIV or PR8 strains. These H1N1 infection models with variable efficiencies of viral clearance result in the accumulation of varying numbers of CCR2+ inflammatrory monocytes, which are highly associated with the generation of a cytokine storm and expression of iNOS. In the early phase of infection, we propose that a small number of infiltratng CCR2+ inflammatory monocytes are infected with IAV and respond to autocrine and/or paracrine IFNβ, which induces the expression of the CCR2 ligands, CCL2, CCL7 and CCL12. Recruited CCR2+ inflammatory monocytes drive further recruitment of CCR2+ inflammatory monocytes from the BM to the lung through CCR2-dependent chemotaxis. In the late phase of infection, impaired clearance of PR8 virus leads to spread of infection to recently arrived CCR2+ inflammatory monocytes and to sustained production of the IFNAR1-IFNβ signaling axis-induced CCR2 ligands, which cause infiltrating CCR2+ inflammatrory monocytes to amplify their own recruitment continuously through the IFNAR1-dependent chemokine feedback loop.

The excessive accumulation of Gr1 + CD11b + cells is strongly associated with severe lung pathology in highly pathogenic 1918 H1N1 and avian H5N1 infections [42]. According to a detailed characterization of Gr1 + CD11b + cells, we found that CCR2+ inflammatory monocytes are a prominent cell type and that they contribute to overabundant inflammatory immune responses. In this study, we demonstrated that the accumulation of infiltrating CCR2+ inflammatory monocytes is determined by the efficiency of host in clearing the virus. Based on our findings, the CCR2+ inflammatory monocytes were one of determinants for pathogenicity of highly pathogenic IAV infection (Figure 8).

## Abbreviations

IAV: Influenza A virus
IFNAR1: interferon-α/β receptor 1
SOIV: swine-origin influenza virus
MCMV: mouse cytomegalovirus
LCMV: lymphocytic choriomeningitis virus
BM: bone marrow
NP: nucleoprotein
MLN: mediastinal lymph node
BALF: bronchoalveolar lavage fluid
CFSE: carboxyfluorescein diacetate succinimidyl ester
NO: nitric oxide
